# Adaptive Multiscale Symbolic-Dynamics Entropy for Condition Monitoring of Rotating Machinery

**DOI:** 10.3390/e21121138

**Published:** 2019-11-21

**Authors:** Chunhong Dou, Jinshan Lin

**Affiliations:** 1 School of Information and Control Engineering, Weifang University, No. 5147 Dong Feng Dong Street, Weifang 261061, China; 2 School of Mechatronics and Vehicle Engineering, Weifang University, No. 5147 Dong Feng Dong Street, Weifang 261061, China

**Keywords:** multiscale, symbolic dynamics, entropy, condition monitoring, rotating machinery

## Abstract

Vibration data from rotating machinery working in different conditions display different properties in spatial and temporal scales. As a result, insights into spatial- and temporal-scale structures of vibration data of rotating machinery are fundamental for describing running conditions of rotating machinery. However, common temporal statistics and typical nonlinear measures have difficulties in describing spatial and temporal scales of data. Recently, statistical linguistic analysis (SLA) has been pioneered in analyzing complex vibration data from rotating machinery. Nonetheless, SLA can examine data in spatial scales but not in temporal scales. To improve SLA, this paper develops symbolic-dynamics entropy for quantifying word-frequency series obtained by SLA. By introducing multiscale analysis to SLA, this paper proposes adaptive multiscale symbolic-dynamics entropy (AMSDE). By AMSDE, spatial and temporal properties of data can be characterized by a set of symbolic-dynamics entropy, each of which corresponds to a specific temporal scale. Afterward, AMSDE is employed to deal with vibration data from defective gears and rolling bearings. Moreover, the performance of AMSDE is benchmarked against five common temporal statistics (mean, standard deviation, root mean square, skewness and kurtosis) and three typical nonlinear measures (approximate entropy, sample entropy and permutation entropy). The results suggest that AMSDE performs better than these benchmark methods in characterizing running conditions of rotating machinery.

## 1. Introduction

Rotating machinery, which plays an important role in modern industry, normally works in complex environments and bears variable loads [1]. As a result, rotating machinery is subject to breakdowns, which may cause a substantial loss [2]. Thus, early detection and prompt treatment of faults is critical to ensuring running safety of machinery [3,4]. In recent decades, the study of this topic has aroused considerable attention. For example, Reference [5] improved multipoint optimal minimum entropy deconvolution adjusted for searching complex fault pulse signals in strong noise environments, Reference [6] designed a simple and fast method for generating enhanced/squared envelope spectra from spectral coherence for fault diagnosis of bearings, Reference [7] developed a novel diagnosis method for locating faults of a bearing outer ring, and Reference [8] presented an intelligent method for fault diagnosis of bearings. Currently, it has been pointed out that vibration data of rotating machinery working in different conditions demonstrate different properties both in spatial scales and in temporal scales [9]. In this sense, insights into spatial- and temporal-scale structures of vibration data of rotating machinery are central to condition monitoring of rotating machinery [1,10,11,12,13,14]. 

Some temporal statistics, such as mean, standard deviation (SD), root mean square (RMS), skewness and kurtosis, have been extensively applied to describe running conditions of machines. Mean, as a static part of data, reflects a global trend of data. As a result, the use of mean seems barely feasible for illustrating complexities of vibration data. SD statistically indicates deviation of data from their mean. Reducing fluctuations of data to only one quantity, SD can simply illuminate a limited part of nature of data. RMS is indicative of energy of data. Unfortunately, RMS displays a low sensitivity to incipient faults of machinery. Skewness measures asymmetry of a probability distribution of data relative to their mean. As a consequence, skewness can convey a part of information contained in complex data. Kurtosis is devised to measure the tailedness of a probability distribution of data. In doing so, kurtosis can describe the shape of probability distribution of data. Accordingly, kurtosis is suitable for detecting impulsive information of data. Nonetheless, a shortage of kurtosis lies in unreliability for different temporal duration. In general, these temporal statistics are effective only for linear and stationary conditions. Therefore, they frequently lose their capability for revealing the nature of nonlinear and nonstationary vibration data gathered from a defective machine. 

The power spectrum, defined as Fourier transform of second-order statistics, usually provides insufficient information about a random signal. Fortunately, higher order spectra (HOS), defined as Fourier transform of higher-order statistics, can serve to capture nonlinear details of a random signal [15]. Among HOS, bispectrum and trispectrum, which represent Fourier transform of third-order statistics and fourth-order statistics, respectively, have found their application in analysis of vibration signals [16]. For example, References [17,18] employed a normalized bispectral measure to examine vibration signals with periodic components and noise, Reference [19] exploited trispectrum for fault diagnosis of rotating machinery, Reference [20] applied HOS to investigate amplitude and phase modulation and Reference [21] used bispectrum to explore a system response. In addition, Reference [22] demonstrated the usefulness of HOS in detecting a fatigue crack of a straight beam and in analyzing vibration signals of rolling bearings, Reference [23] displayed the potential of bispectrum and trispectrum for fault diagnosis of rotating machinery, Reference [24] made use of HOS to distinguish between cracks and misalignment in a rotating shaft and Reference [25] made a comparison between the results of HOS and higher order coherence for fault diagnosis of rotating machinery. However, HOS performs unequally well in deterministic and nondeterministic cases and may produce obscure spectra for a narrowband signal [26]. Additionally, results acquired by HOS generally lack clear physical meaning [17]. Furthermore, HOS seemingly lacks the ability to describe spatial- and temporal-scale structures of vibration signals. 

Several widespread nonlinear measures, that is, approximate entropy (ApEn), sample entropy (SaEn) and permutation entropy (PeEn), have been proposed for examining complexities of data [27,28,29]. ApEn can measure complexities and regularity of data and has the potential to analyze short and noisy data [27,30]. Nonetheless, ApEn is beset with two deficiencies [31]. Firstly, owing to high sensitivity to the size of data, ApEn generally deviates from real nature of data when applied to probe small-size data [31]. Secondly, ApEn appears inconsistent across all the conditions [31]. To this end, SaEn was developed for refining ApEn [31]. Compared with ApEn, SaEn shows higher computational efficiency and better consistency across all the conditions [31]. Unfortunately, SaEn still demonstrates a limited ability to investigate dynamics of data. Afterwards, PeEn was proposed for revealing dynamics of noisy data [32]. In PeEn, original data in a fixed-length window are firstly translated into symbols and then occurrence of every possible permutation of the symbols boils down to one quantity. Unlike information entropy, fractal dimension and the Lyapuaov exponent, which are designed specially to analyze ergodic random variables, PeEn is suitable for processing any type of data. Compared with nonlinear monotonous transformation, PeEn delivers a better performance in computational efficiency, robustness and invariance [32]. Nevertheless, PeEn, along with ApEn and SaEn, shows spatial-scale structures of complex data but neglect their temporal-scale structures [33]. As a consequence, these shortages seriously limit the spread of these nonlinear measures. As stated above, PeEn is an entropy measure based on symbolic dynamics. This suggests that symbolic dynamics has the potential to disclose useful repetitions buried in original data [34]. Currently, symbolic dynamics has blossomed into an effect method for data analysis [35]. For example, Reference [36] related visibility graphs to symbolic dynamics and Reference [37] combined modified multi-scale symbolic dynamic entropy with max-relevance and min-redundancy (mRMR) features for fault diagnosis of planetary gearboxes. The basic principle of symbolic dynamics is to convert original data into several symbols through a coarse-graining rule and to dig up information contained in these symbols. Therefore, symbolic dynamics has the capability to expose robustness and invariance of complex data by neglecting trivial details of these data [38]. Accordingly, symbolic dynamics can provide a deep insight into the nature of complex data. Unfortunately, traditional coarse-graining rules are barely adaptive due to dependence of a model containing invariable thresholds [37]. Our early work has developed an adaptive statistical linguistic analysis (SLA) for investigating vibration signals of machines [39]. SLA can transform original data into a binary symbolic series according to increased or decreased relationships between two consecutive elements in original data, without any presetting. Moreover, by choosing a specific temporal scale, a binary symbolic series can be mapped to a set of word types. Next, dynamics of original data are analyzed by examining occurrence of every word type. Nevertheless, SLA is confronted by the following three problems. Firstly, it is hard and awkward for SLA to choose an appropriate temporal scale. Secondly, a word-frequency series yielded by SLA is barely concise in describing running conditions of rotating machinery. Thirdly, SLA can examine data in spatial scales but not in temporal scales. The idea of PeEn motivated us to remedy these deficiencies of SLA. In this respect, this paper firstly develops a novel concept of symbolic-dynamics entropy by reducing a word-frequency series to one quantity. Also, this paper introduces multiscale analysis to SLA to gain an insight into temporal scales of data. As a result, this study proposed adaptive multiscale symbolic-dynamics entropy (AMSDE). By AMSDE, spatial- and temporal-scale structures of data can be disclosed by a set of symbolic-dynamics entropy, each of which refers to a specific temporal scale. Next, this study exploited AMSDE to investigate vibration signals collected from defective gearboxes and rolling bearings. Additionally, the performance of AMSDE was benchmarked against these five common temporal statistics, i.e., mean, standard deviation (SD), root mean square (RMS), skewness and kurtosis, and these three typical nonlinear measures, i.e., ApEn, SaEn and PeEn. The results indicated that AMSDE exhibits adequate reliability and has an advantage over these benchmark methods in distinguishing between different running conditions of rotating machinery. 

This paper is structured below. Section 2 formulates AMSDE. Section 3 compares AMSDE with some prevailing temporal statistics and nonlinear measures and opens up a detailed discussion about the results. Finally, Section 4 comes to a conclusion.

## 2. Adaptive Multiscale Symbolic-Dynamics Entropy (AMSDE)

### 2.1. Adaptive Coarse-Graining Algorithm

Intrinsic fluctuations of data from a dynamical system convey a lot of information on dynamics of the system. Indeed, an increase or a decrease between two consecutive elements of original data is dominated by dynamical mechanism of a system [40,41,42]. For example, these increasing or decreasing features have been successfully exacted to expose physiologic dynamics in Reference [40], to characterize linguistic styles of different authors in Reference [41] and to describe human rate series and DNA sequences in Reference [42]. Thus, quantification of these increases or decreases can serve to reflect dynamics of a system [39]. If an increase and a decrease between two consecutive elements of original data are represented by 1 and 0, respectively, original data can be translated into a binary symbolic series. Hence, for a series $x_{i}$(i=1, 2,…, N), a binary symbolic series $bss_{i}$(i=1, 2,…, N−1) is defined as

(1) $$bss_{i}=\{\begin{array}{ccc} 0 & x_{i}\geq x_{i+1} & i=1,\;2,\ldots ,\;N-1\\ 1 & x_{i}<x_{i+1} & i=1,\;2,\ldots ,\;N-1 \end{array}$$

### 2.2. AMSDE

Define *m* successive binary symbols as an *m*-bit word. Here, the parameter *m* is called a temporal scale. By moving one symbol at a time, a binary symbolic series can be translated into a word series. As can be calculated, a temporal scale *m* can produce (N−m+1) words, which contains at most $2^{m}$ types of word. By documenting the occurrence of every word type, one can derive a word-frequency series [39]. 

To exhibit temporal-scale structures of data, multiscale analysis is introduced to SLA by varying the temporal scale in a limited range. Supposing that the temporal scale *m* takes a value from $\left[m_{1},m_{2},\ldots ,m_{k}\right]$, when $m=m_{j}\left(j\leq k\right)$, one obtains a word-probability series $\left[p_{1}\left(m_{j}\right),p_{2}\left(m_{j}\right),\ldots ,p_{n}\left(m_{j}\right)\right]$,

(2) $$p_{i}\left(m_{j}\right)=\frac{N_{i}}{\sum_{i=1}^{n}N_{i}},\;n\leq 2^{m_{j}}$$

Here, $N_{i}$ and $p_{i}\left(m_{j}\right)$ stand for the frequency and the probability of occurrence of the *i*th word type for temporal scale $m_{j}$, respectively. Next, the symbolic-dynamics entropy for temporal scale $m_{j}$ is defined as

(3) $$E\left(m_{j}\right)=-\sum_{i=1}^{n}\left\{p_{i}\left(m_{j}\right)\log\left[p_{i}\left(m_{j}\right)\right]\right\}$$

Afterwards, AMSDE is represented as a set of $\left[E\left(m_{1}\right),E\left(m_{2}\right),\ldots ,E\left(m_{k}\right)\right]$ by collecting all the symbolic-dynamics entropy for different temporal scales.

## 3. Application of AMSDE to Condition Monitoring of Rotating Machinery

### 3.1. Condition Monitoring of Gears

**Figure 1:**
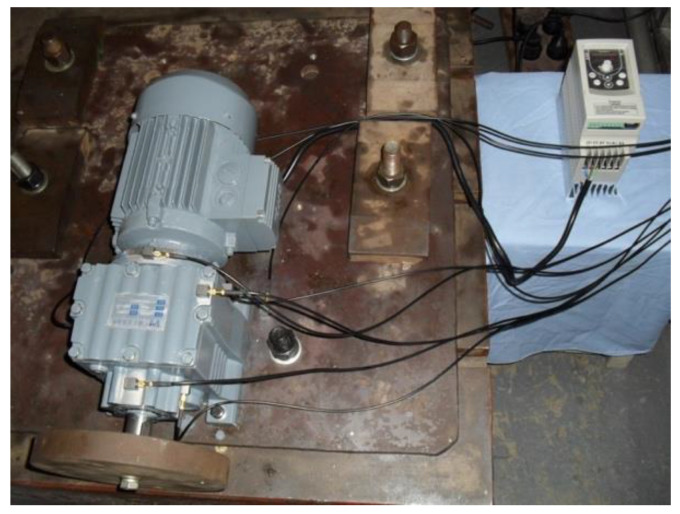
An experimental rig of simulating gear faults.

**Figure 2:**
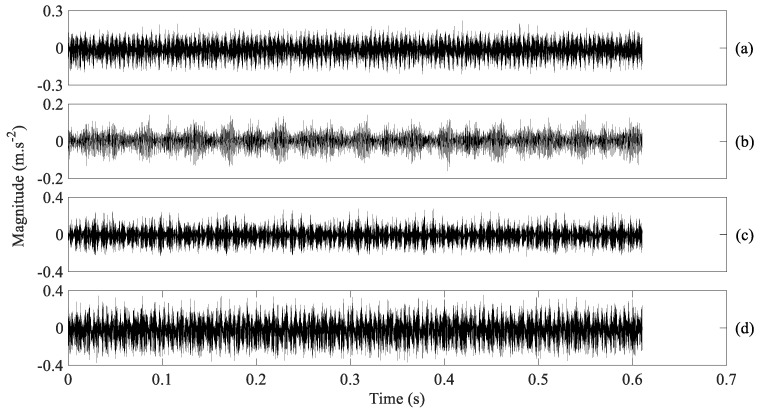
Four types of gearbox vibration signals, (**a**–**d**) for normal, slight-scratch, medium-scratch and broken-tooth gear conditions, respectively.

The performance of AMSDE was tested using vibration signals from a defective gearbox of two-stage transmission. An experimental rig for simulating gear faults is depicted in Figure 1. The gearbox (Autofast Technologies Co., Ltd., Taizhou, China) was fixed on an experimental table and driven by an AC motor(Autofast Technologies Co., Ltd., Taizhou, China) with a revolving speed of 2000 revolutions per minute (RPM). This gearbox experiment simulated four types of gear conditions: normal, slight-scratch, medium-scratch and broken-tooth. Here, a considerable difficulty lies in distinguishing between slight-scratch and medium-scratch, which are similar. Sixteen segments of vibration signals were recorded for each gear condition, each segment with a sampling frequency of 16,384 Hz and a size of 10,000 points. These four types of gearbox vibration signals are described in Figure 2. 

**Figure 3:**
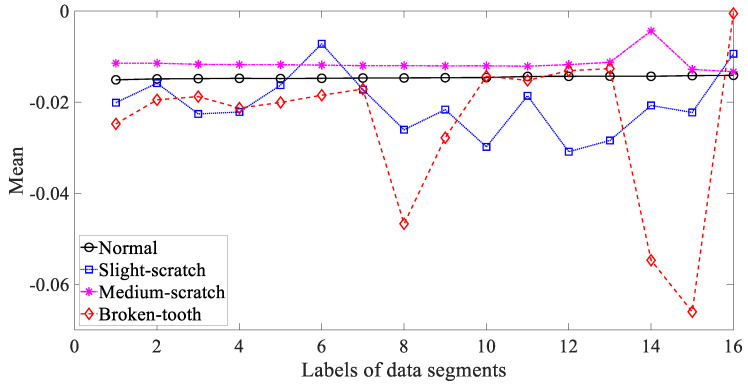
Comparisons between mean for four types of gear condition.

**Figure 4:**
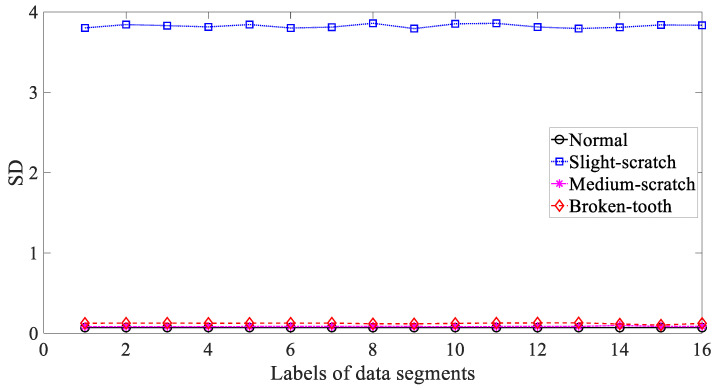
Comparisons between standard deviation (SD) for four types of gear condition.

**Figure 5:**
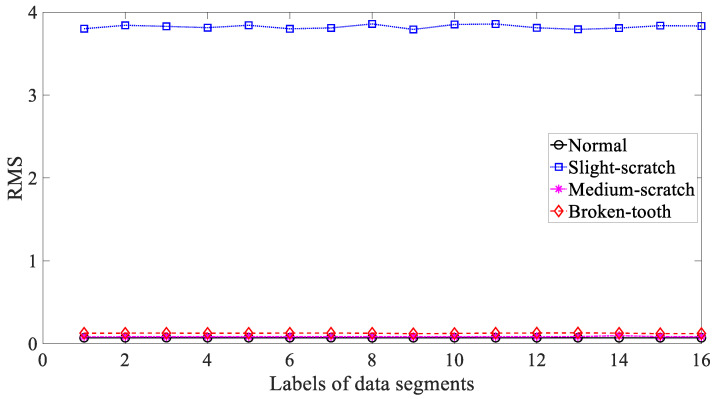
Comparisons between root mean square (RMS) for four types of gear condition.

**Figure 6:**
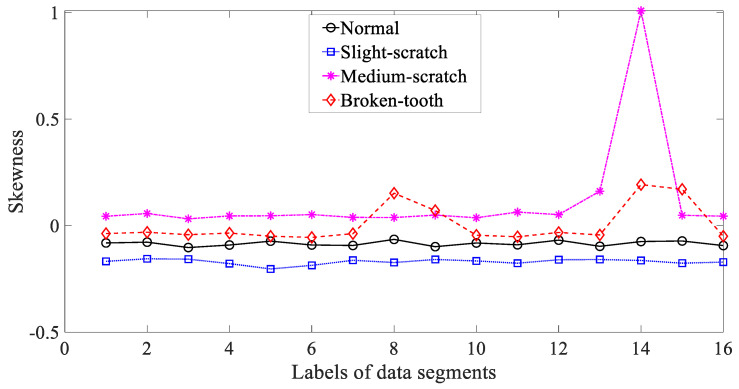
Comparisons between skewness for four types of gear condition.

**Figure 7:**
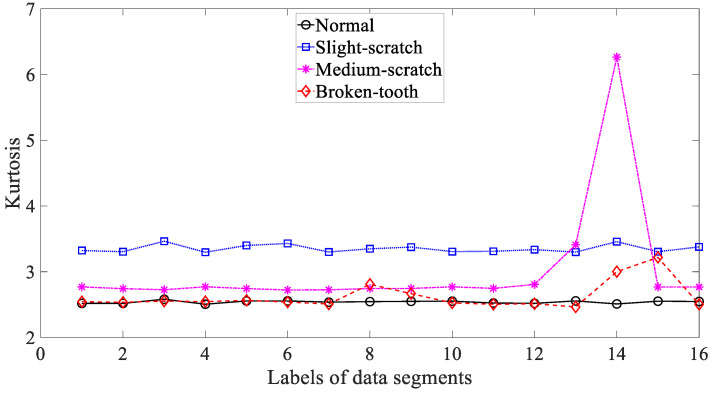
Comparisons between kurtosis for four types of gear condition.

Five common temporal statistics, i.e., mean, SD, RMS, skewness and kurtosis, were employed to investigate these gearbox vibration signals. Firstly, mean was employed to describe these four types of gear condition and the results are given in Figure 3. As given in Figure 3, the mean for slight-scratch and broken-tooth fluctuated greatly. In addition, the mean for these four types of gear condition intersected severely. Therefore, it followed that the mean was unreliable for monitoring running conditions of the gear. Next, SD was applied to investigate these gearbox vibration signals and the results are exhibited in Figure 4. As exhibited in Figure 4, there was a severe intersection between SD for normal, medium-scratch and broken-tooth conditions. Consequently, this suggested that SD demonstrated a limited ability to depict running conditions of the gear. Furthermore, RMS was adopted to analyze these gearbox vibration signals and the results are shown in Figure 5. As shown in Figure 5, RMS for normal, medium-scratch and broken-tooth conditions intersected severely. As a consequence, this indicated that RMS lacked the capability to portray running conditions of the gear. Also, a similarity between SD in Figure 4 and RMS in Figure 5 for these four types of gear condition suggested that these gearbox vibration signals were zero-mean. Moreover, the use of skewness was made to examine these gearbox vibration signals and the results are given in Figure 6. As given in Figure 6, skewness for medium-scratch experienced large fluctuations. Additionally, skewness for medium-scratch and broken-tooth intersected locally. This proves that skewness hardly undertook a task for characterizing running conditions of the gear. Afterwards, kurtosis demonstrated its use in investigating these gearbox vibration signals and the results are illustrated in Figure 7. As illustrated in Figure 7, kurtosis for medium-scratch had considerable fluctuations. Also, there were many intersections between kurtosis for these four types of gear condition. This means that kurtosis lacked the capability for depicting running conditions of the gear. 

**Figure 8:**
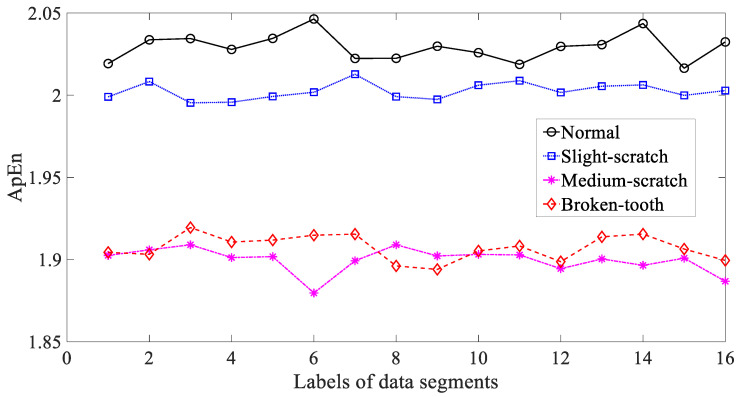
Comparisons between approximate entropy (ApEn) for four types of gear condition.

**Figure 9:**
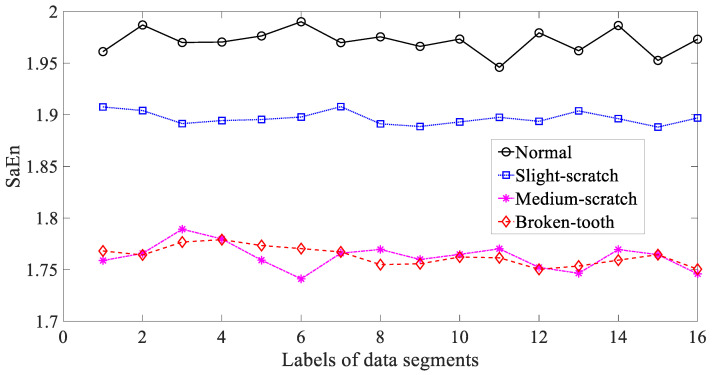
Comparisons between sample entropy (SaEn) for four types of gear condition.

**Figure 10:**
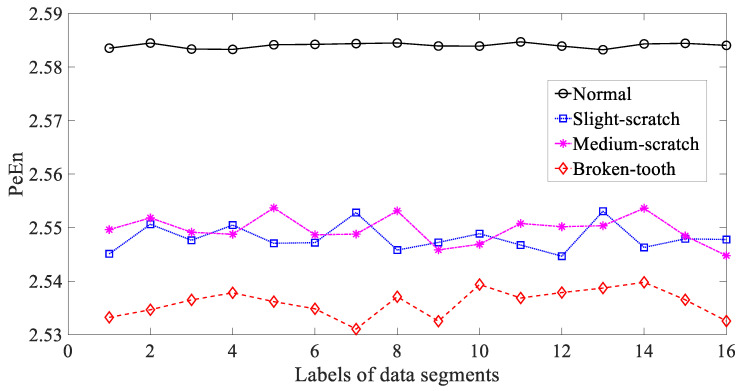
Comparisons between permutation entropy (PeEn) for four types of gear condition.

In the following, three typical nonlinear measures, i.e., ApEn, SaEn and PeEn, were adopted to investigate these gear vibration signals. Firstly, ApEn was employed to study these gear vibration signals and the results are exhibited in Figure 8. As exhibited in Figure 8, ApEn for these four types of gear condition had some small fluctuations. Additionally, ApEn for medium-scratch and broken-tooth intersected locally. This indicated that ApEn was not entirely dependable in representing running conditions of the gear. Next, SaEn was put in use for researching these gearbox vibration signals and the result are demonstrated in Figure 9. As demonstrated in Figure 9, SaEn for normal and medium-scratch conditions had small fluctuations. Moreover, SaEn for medium-scratch and broken-tooth intersected severely. This means that SaEn lacked enough reliability for depicting running conditions of the gear. In addition, PeEn was applied to probe these gearbox vibration signals and the results are displayed in Figure 10. As displayed in Figure 10, PeEn for slight-scratch, medium-scratch and broken-tooth had small fluctuations. Additionally, PeEn for slight-scratch and medium-scratch intersected severely. This suggested that PeEn failed to exhibit entirely feasibility for describing running conditions of the gear. 

**Figure 11:**
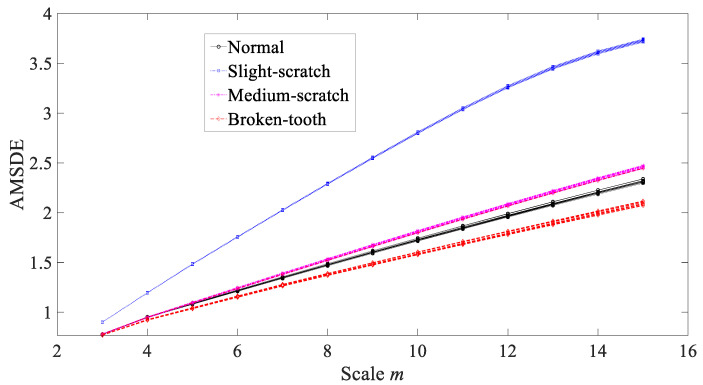
Comparisons between adaptive multiscale symbolic-dynamics entropy (AMSDE) for four types of gear condition.

Finally, AMSDE was exploited to process these gearbox vibration signals and the results are revealed in Figure 11. As revealed in Figure 11, with good reliability for each gear condition, AMSDE delivered an excellent performance in distinguishing between these four types of gear condition. 

### 3.2. Condition Monitoring of Rolling Bearings

**Figure 12:**
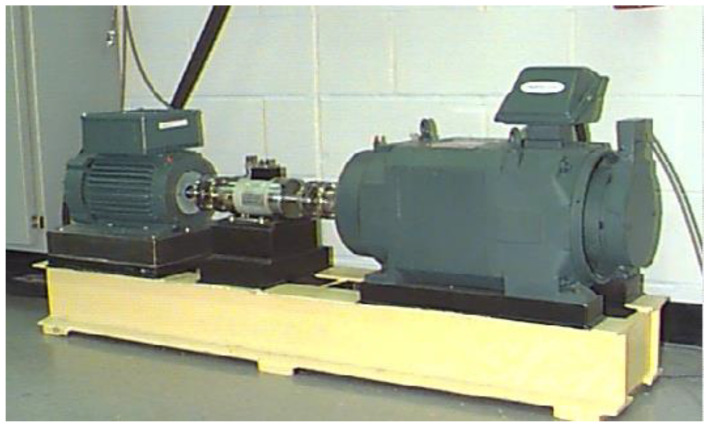
An experimental rig for simulating rolling-bearing faults.

**Figure 13:**
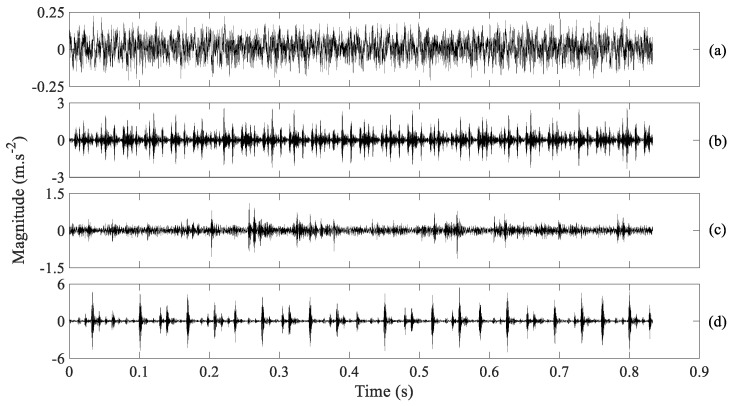
Four types of rolling-bearing vibration signals, (**a–d**) for normal, inner-race faults, ball faults and outer-race faults, respectively.

The performance of AMSDE was further measured using rolling-bearing vibration signals from the Case Western Reserve University Bearing Data Center Website [43]. The rolling-bearing experiment, described in Figure 12, simulated four types of bearing conditions: normal, inner-race faults, ball faults and outer-race faults. The revolving speed of a driving motor fluctuated between 1797 RPM and 1720 RPM. Twelve segments of data were gathered for each bearing condition, each segment with a sampling frequency of 12,000 Hz and a size of 10,000 points. These four types of bearing vibration signals are profiled in Figure 13. 

**Figure 14:**
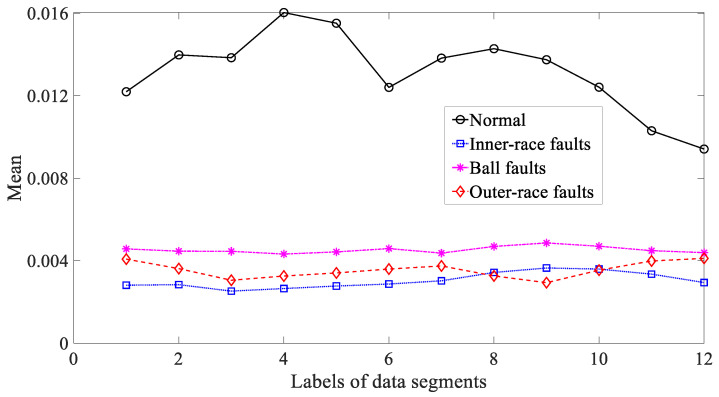
Comparisons between mean for four types of rolling-bearing condition.

**Figure 15:**
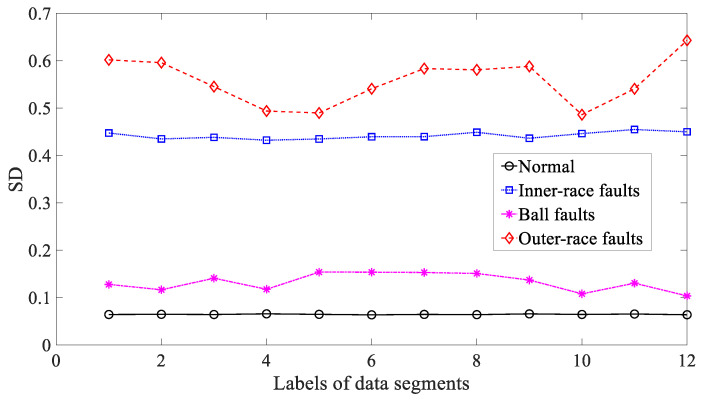
Comparisons between standard deviation (SD) for four types of rolling-bearing condition.

**Figure 16:**
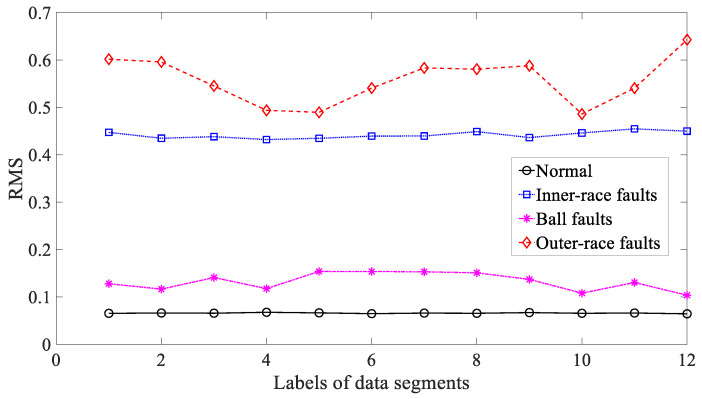
Comparisons between root mean square (RMS) for four types of rolling-bearing condition.

**Figure 17:**
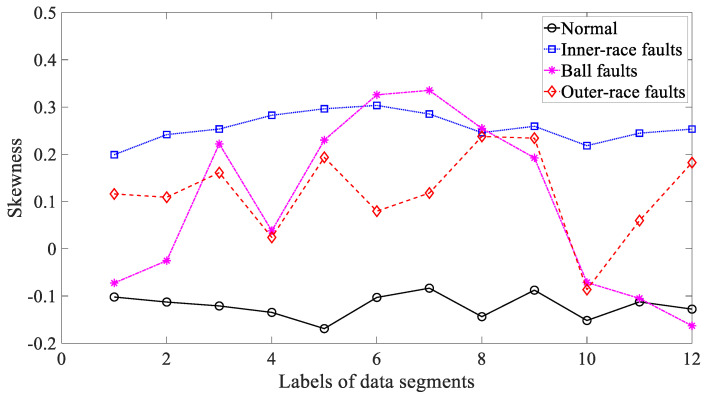
Comparisons between skewness for four types of rolling-bearing condition.

**Figure 18:**
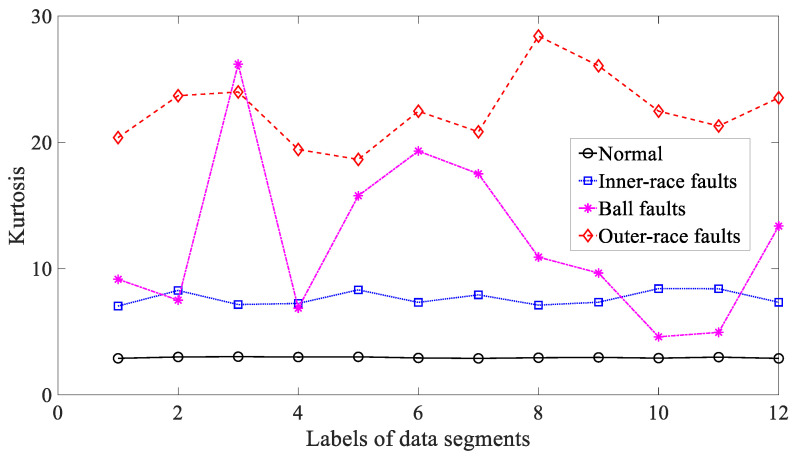
Comparisons between kurtosis for four types of rolling-bearing condition.

Five common temporal statistics, i.e., mean, SD, RMS, skewness and kurtosis, were employed to examine these bearing vibration signals. To begin with, mean was used to explore these bearing vibration signals and the results are reflected in Figure 14. As reflected in Figure 14, mean for inner-race, ball and outer-race faults intersected locally. This implied that mean was not a dependable parameter descriptive of running conditions of the bearing. Next, SD was applied to study these bearing vibration signals and the results are revealed in Figure 15. Although successful in discriminating between these four types of bearing condition, as revealed in Figure 15, SD for outer-race faults fluctuated greatly. Then, RMS was adopted to probe these bearing vibration signals and the results are illustrated in Figure 16. These bearing vibration signals have a zero-mean property, therefore RMS in Figure 16 resembles SD in Figure 15. This means that RMS and SD were scarcely capable of characterizing running conditions of the bearing. Subsequently, skewness illustrated its usefulness in examining these bearing vibration signals and the results are provided in Figure 17. As provided in Figure 17, skewness for ball and outer-race faults fluctuated dramatically. Additionally, skewness for these four types of bearing condition intersected severely. This hinted that skewness performed very poorly in giving a description of running conditions of the bearing. Next, kurtosis came to these bearing vibration signals and the results are displayed in Figure 18. As displayed in Figure 18, kurtosis for ball and outer-race faults fluctuated dramatically. In addition, kurtosis for inner-race, ball and outer-race faults intersected severely. This denoted that kurtosis was hard to tackle the task for depicting running conditions of the bearing. 

**Figure 19:**
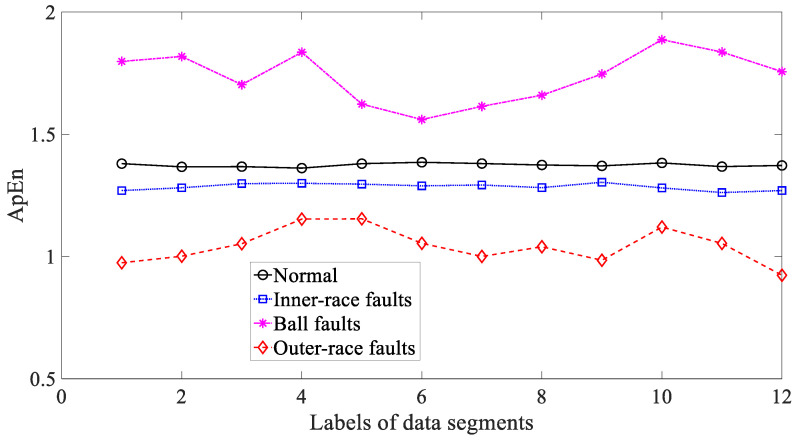
Comparisons between approximate entropy (ApEn) for four types of rolling-bearing condition.

**Figure 20:**
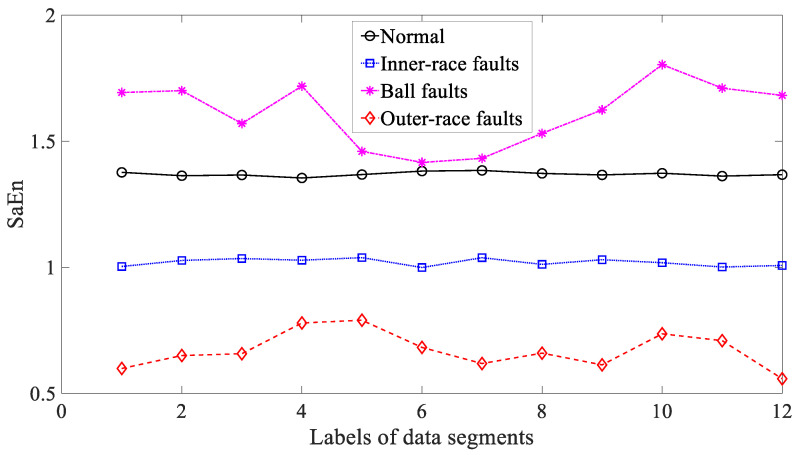
Comparisons between sample entropy (SaEn) for four types of rolling-bearing condition.

**Figure 21:**
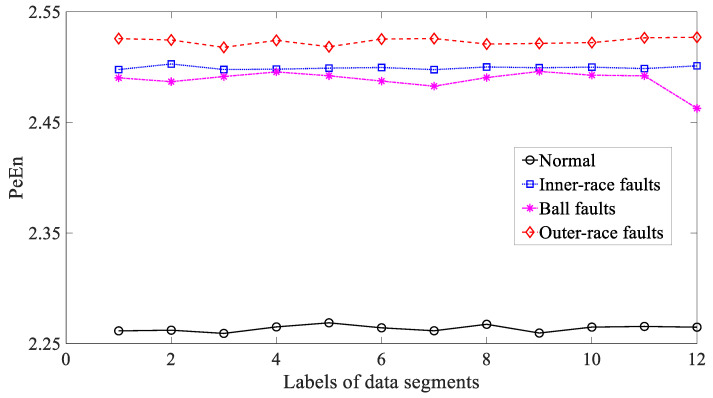
Comparisons between permutation entropy (PeEn) for four types of rolling-bearing condition.

Afterwards, three typical nonlinear measures, i.e., ApEn, SaEn and PeEn, were used to analyze these bearing vibration signals. Firstly, ApEn was employed to investigate these bearing vibration signals and the results are exhibited in Figure 19. Although capable of distinguishing between these four types of bearing condition, as exhibited in Figure 19, ApEn for ball and outer-race faults demonstrated large fluctuations. This gives evidence that ApEn had insufficient reliability for characterizing running conditions of the bearing. Moreover, SaEn displayed its application in processing these bearing vibration signals and the results are displayed in Figure 20. As displayed in Figure 20, SaEn for ball and outer-race faults held large fluctuations. This indicated that SaEn was insufficiently stable in describing running conditions of the bearing. In the following, PeEn was adopted to analyze these bearing vibration signals and the results are shown in Figure 21. As shown in Figure 21, it seemed hard for PeEn to distinguish between inner-race and ball faults. This meant that PeEn was not always effective in depicting running conditions of the bearing. 

**Figure 22:**
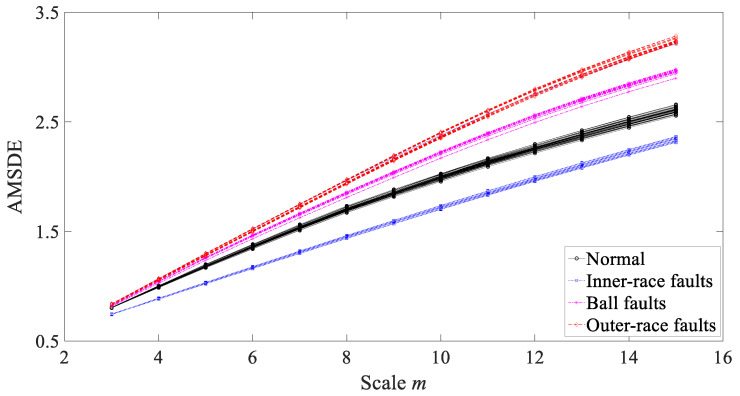
Comparisons between adaptive multiscale symbolic-dynamics entropy (AMSDE) for four types of rolling-bearing condition.

Then, AMSDE was exploited to investigate these bearing vibration signals and the results are exhibited in Figure 22. As exhibited in Figure 22, AMSDE demonstrated excellent reliability for describing running conditions of the bearing and performed well in distinguishing between these four types of bearing condition. 

### 3.3. Results and Discussions

AMSDE was used to examine vibration signals from both defective gearboxes and rolling bearings. As a result, the symbolic-dynamics entropy increased with a temporal scale, as shown in Figure 11 and in Figure 22. This manifests that types of a large-bit word distribute more equally than those of a small-bit word. Therefore, this offers a proof that these vibration signals display multiscale properties. Moreover, the performance of AMSDE was benchmarked against these five common statistics and three typical nonlinear measures. The results indicated that AMSDE delivered a better performance in describing running conditions of rotating machinery and had a clear advantage over these benchmark methods. 

This paper makes two main contributions. Firstly, this paper defined the symbolic-dynamics entropy for quantifying probability distributions of word types for a specific scale. The symbolic-dynamics entropy defined in this paper could reduce a large-size word-frequency series to one quantity, which has the potential to express essence of original data. In fact, this simplification enabled SLA to be directly compared with some widespread statistics. Secondly, AMSDE was proposed by introducing multiscale analysis to SLA. Indeed, vibration signals of rotating machinery displayed distinctly different structures in different temporal scales. Therefore, it was necessary to introduce multiscale analysis to SLA. As a matter of fact, the introduction of multiscale analysis has two potential advantages. For one thing, it can avoid difficulties, which SLA faces, in choosing an optimal temporal scale. For another, it makes up deficiencies of SLA, which investigates original data only in a given temporal scale. As a consequence, AMSDE can demonstrate structures of data in both spatial and temporal scales.

Although exhibiting interesting features, AMSDE still encounters several problems. To begin with, a mechanism that the symbolic-dynamics entropy varies with a change of running conditions of rotating machinery currently seems unclear and waits to be investigated in the future. In addition, the feasibility and reliability of AMSDE requires a more convincing demonstration using extensive data from various types of machine. 

## 4. Conclusions

This study introduced multiscale analysis to symbolic dynamics for overcoming deficiencies of SLA and proposed AMSDE for describing running conditions of rotating machinery. Afterwards, AMSDE was adopted to examine vibration signals from defective gearboxes and rolling bearings. Also, AMSDE was compared with five common statistics, and three typical nonlinear measures. The results showed that AMSDE demonstrated good reliability in describing running conditions of rotating machinery and was superior to these benchmark methods in distinguishing between different running conditions of rotating machinery.
